# Implementation of the Integrated Management of Childhood Illnesses strategy: challenges and recommendations in Botswana

**DOI:** 10.3402/gha.v9.29417

**Published:** 2016-02-17

**Authors:** Lucia U. Mupara, Johanna C. Lubbe

**Affiliations:** 1 Department of Health Studies, University of South Africa, Pretoria, South Africa; 2 School of Public Health, Department of Health Promotion and Education, Boitekanelo College, Gaborone, Botswana

**Keywords:** IMCI, implementation challenges, registered nurses, children under 5 years, Millennium Development Goals

## Abstract

**Background:**

Under-five mortality has been a major public health challenge from time immemorial. In response to this challenge, the World Health Organization and the United Nations Children's Fund developed the Integrated Management of Childhood Illnesses (IMCI) strategy and presented it to the whole world as a key approach to reduce child morbidity and mortality. Botswana started to implement the IMCI strategy in 1998. Reductions in the under-five mortality rate (U5MR) have been documented, although the reduction is not on par with the expected Millennium Development Goal 4 predictions.

**Design:**

A quantitative study was done to identify the problems IMCI implementers face when tending children under 5 years in the Gaborone Health District of Botswana. The study population was made up of all the IMCI-trained and registered nurses, and systematic sampling was used to randomly select study participants. Questionnaires were used to collect data.

**Results:**

The study findings indicated challenges related to low training coverage, health systems, and the unique features of the IMCI strategy.

**Conclusions:**

The comprehensive implementation of the IMCI strategy has the potential to significantly influence the U5MR in Botswana.

## Introduction

Santana sings, ‘Let the children play’, but in our civilised world children are dying without ever having had a chance to play. Globally, every 3 seconds a child dies (1). In the African region alone, 12,000 children between the ages of 0 and 5 years die on a daily basis (2). This calculates to a global under-five mortality rate (U5MR) of 29,000 per day and an African U5MR of nearly 4.5 million per annum! Newborns have become the ‘forgotten children of Africa’ (2).

In sub-Saharan Africa, children under the age of five are 15 times more likely to die, compared to children in developed countries (3). Botswana is no different; 5.67% of all children born in Botswana will never reach their first birthday. An alarming 7.4% of these children will not reach their fifth birthday (4, 5). This is a heart-breaking situation for every grandparent, parent, and nurse, given the fact that many of these deaths are preventable. The World Health Organization (WHO) reports that 70% of child deaths are caused by five preventable and treatable diseases or conditions, namely acute respiratory infections (mostly pneumonia), diarrhoea, measles, malaria, and malnutrition (PDMMM) and often a combination of these conditions (3). The United Nations Millennium Development Goal 4 was specifically developed to prevent these deaths, but despite all efforts children are still dying from preventable diseases.

The 1996 analysis published under the title ‘The global burden of disease’ (6) projected that the above five killer conditions will continue to be major contributors to child deaths by the year 2020, unless significantly greater efforts are made to control them (6). These killer conditions will still account for 52% of under-five deaths by 2020, unless efforts are accelerated to address them.

In line with the United Nations Millennium Development Goals (MDGs) (7), Botswana developed its Vision 2016, which articulates the country's developmental aspiration, Towards Prosperity for All. This vision is guided by seven pillars, which articulate the MDGs. Vision 2016's third pillar, which seeks to build a compassionate, just, and caring nation, cites health, among other things, as one of its priorities. It is within this third pillar that MDG4 is addressed. MDG4 focuses on child mortality, which has been a major public health challenge from time immemorial.

The government of Botswana, through the Ministry of Health (MoH), introduced the Accelerated Child Survival and Development (ACSD) strategic plan intervention with a specific focus on reducing the U5MR. One of the high-impact interventions for reducing the U5MR is the Integrated Management of Childhood Illness (IMCI) strategy. The IMCI strategy was formulated by the WHO and the United Nations Children's Fund (UNICEF) and was presented to the world in 1996 as the principal strategy to reduce child mortality and improve child health (2, 8). More than 100 countries adopted the IMCI strategy (9).

The WHO (2) defines the IMCI as a strategy formulated by the WHO and UNICEF; it was presented in 1996 as the supreme strategy for improving child health. IMCI strategy presents a unique public health opportunity, in that it is an integrated approach to child health that focuses on the well-being of the whole child. IMCI aims to reduce death, illness, and disability and to promote improved growth and development among children under 5 years of age. IMCI includes both preventive and curative elements, which are implemented by families and communities, as well as by health facilities. Thus, it reduces missed opportunities for early detection and treatment of diseases, which can escape the notice of both parents and health workers (4).

The main goal of IMCI is to contribute to healthy growth and development, during the first 5 years of life. This goal comprises three objectives:

- Reducing infant mortality
- Reducing the incidence and seriousness of illnesses and health problems that affect boys and girls
- Improving growth and development during the first 5 years of a child's life (10, 11)

The IMCI strategy consists of three components (11). The first is the health worker component. This component seeks to improve the case management skills of health workers who are tending patients under 5 years old. It is implemented through integrated case management, founded on case detection, by using simple clinical signs and experiential treatment (2). The integrated case management process was designed for doctors, nurses, and other health professionals who are tending sick infants and children aged 0 to 5 years. It is an evidence-based, case management process for a first-level facility such as a clinic, a health centre, or an outpatient department of a hospital (2).

**Fig. 1:**
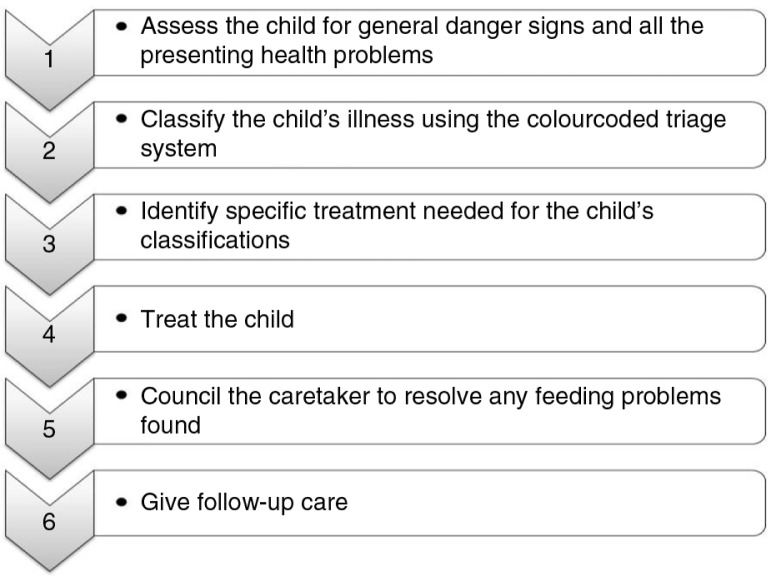
The Integrated Management of Childhood Illnesses integrated case management process.

The second component entails the health system or service component (11). It targets the improvement of the organisation and overall functioning of the healthcare system (i.e. to offer efficient and effective management of childhood illnesses). The complete IMCI case management process involves the following elements, as illustrated in Fig. 1.

The community component, as the third component (11), seeks to improve family and community practices that have direct impact on the health of under-five children.

## Progress in Botswana

According to the IMCI Health Facility Survey (HFS) (12), the IMCI strategy was introduced to Botswana in 1997. The IMCI working group was formed in 1997, and 5 (out of 24) districts were selected to start IMCI implementation. A national orientation workshop followed to advocate the impact of the strategy in reducing mortality rates among infants and children under five. It also recorded that by 1998 the generic materials were adapted, and in 1999 national IMCI training was conducted.

The IMCI HFS (12) reported that by 2004, IMCI was part of the national health delivery system, with government commitment and funding of US$180,000 per year, as well as the support of partners such as the WHO and UNICEF. Moreover, by 2004 IMCI was implemented in 11 of the 24 districts of Botswana. By December 2010, the WHO published a *Child and Adolescent Health Annual Report* (13), which reported that Botswana had reached the expansion phase, since 75% of its health districts were implementing the IMCI strategy. In addition, Botswana was also reported to have received technical support for IMCI pre-service training from the WHO (3).

According to the Botswana Demographic Survey, the 2005 infant mortality rate and the U5MR in Botswana experienced 51 and 76 deaths, respectively, per 1,000 live births (14). Five years later (i.e. in 2010), the United Nations indicated success where the U5MR declined to 36.1/1,000 live births in Botswana (15). Furthermore, the ACSD Strategy Plan for 2009/2010–2015/2016 (16) reported that Botswana attained high coverage of most child survival interventions. The ACSD Strategy Plan stated that 90% of children were fully immunised at the age of one, 97% of pregnant women attended antenatal care clinics, and 98.5% of deliveries took place with skilled attendants. The report also indicated that Botswana had led the way in the implementation of the prevention of mother-to-child transmission of HIV programme, with 89% of HIV-infected pregnant women receiving antiretroviral drugs. Currently, the government of Botswana gives free immunisations, medical treatment, and food (formula and porridge) to all children under the age of five (16). There has been ongoing IMCI-1 training of health service workers in Botswana since 1999, continuing to the present.

However, despite all these successful measures in place, the U5MR in Botswana is still not on par with MDG4 projections. Furthermore, Botswana's ACSD Strategy Plan for 2009/2010–2015/2016 (16) revealed that the same five preventable and treatable conditions (PDMMM) are still the leading causes of child mortality.

The above statistics and predictions clearly indicate that although progress was made, certain deficits still remain. The authors were unable to find documented research findings that identify possible challenges in the implementation of the IMCI strategy by the IMCI-trained registered nurses; hence they undertook this study to identify the challenges faced by IMCI-trained, registered nurses in implementing the set guidelines and procedures of this strategy when tending children under 5 years of age. The findings could assist policymakers and health planners to address problems identified. Addressing the challenges experienced by the registered nurses could contribute to a further lowering of child mortality in Botswana and other African countries.

## Methods

An explorative, descriptive, quantitative study with qualitative enhancement was conducted to identify challenges faced by the IMCI-1-trained, registered nurses in the Gaborone district when implementing the guidelines and procedures established by the IMCI-1 strategy. The target population was all the registered nurses (*N=*90) in the Gaborone Health District who were trained in the health worker component of the IMCI strategy and were tending children under 5 years of age in their current station of duty.

## Authenticity

An extensive literature review was conducted to identify authentic topics for survey questions. This step was followed by consultations with experts from the WHO and UNICEF. Members from both groups evaluated the data-gathering instrument to ensure stability, equivalence, homogeneity, validity, and reliability. The researchers also collaborated with the national IMCI coordinator, the IMCI national focal person, and the medical practitioner responsible for IMCI-1-trained registered nurses in the Gaborone Health District.

A pilot study was conducted using participants who met the inclusion criteria. This was done to establish consistency of response, as well as the validity and reliability of the instrument. During the 2-week data collection period, the researcher carefully monitored the environment to ensure that the conditions under which data were collected were homogenous.

### Data gathering

Using Slovin's formula [*n*=*N*/(1 + *Ne* ^2^)], the sample size (*n*), with a confidence level of 90% and a margin error of 10%, calculated to 39 participants. Systematic sampling was performed and a slightly larger sample of 45 participants was randomly selected from all of the 15 clinics in Gaborone Health District where the IMCI-trained nurses were deployed.

A structured questionnaire was used to extract information about the possible challenges faced by the IMCI-trained, registered nurses in implementing the IMCI strategy and to solicit relevant recommendations on how to improve the implementation of this strategy. About 32 participants completed the questionnaire. Data from the closed questions were statistically analysed. Descriptive statistics such as frequency distributions, measures of central location, and measures of dispersion were used to describe and summarise the data.

## Results and discussion

The results were originally statistically analysed. However, they were also thematised and categorised as illustrated in Table 1. The rationale for this qualitative augmentation of the quantitative results was to convert the data into a format that could be used to collate results and to enable the researchers to use the data to make tangible recommendations that can be implemented in a primary healthcare setting.

**Table 1:** Implementation challenges and recommendations

Theme	Sub-theme	Recommendations
Features of the IMCI strategy	• IMCI consultations are time-consuming • IMCI leads to longer waiting queues • Lack of IMCI implementation resources	• Increase the number of IMCI-trained implementers through the following measures: ° Addressing the problem of understaffing ° Improving the patient–nurse ratio ° Advocating for more resources
Training coverage	Low training coverage	• Increase the number of health workers able to deliver IMCI services by: ° Scaling up both pre-service and in-service IMCI training ° Extending IMCI training to lower-level cadres ° Providing compulsory training for all nurses tending children under 5 years of age
Health system	Physical layout of facilities	• Provide a facility layout that allows for space and time to apply IMCI skills and procedures
	Supervisor support	• Train senior managers in the IMCI strategy to boost their confidence in supervising IMCI protocol implementers • Increase the number of IMCI facilitators
	Follow-up training and visits	• IMCI facilitators should undertake IMCI follow-up training and visits to IMCI implementers
Client expectations	Client expectations	• Standardisation of case management practices of all registered nurses tending children under five

IMCI, Integrated Management of Childhood Illnesses.

The three themes identified indicated specific challenges related to the unique features of the IMCI strategy, low training coverage, and health system and client expectations.

### Challenges related to the unique features of the IMCI strategy

Challenges related to the unique features of IMCI were that IMCI consultations are time-consuming and lead to longer patient-waiting queues. If the queues get too long, the non-IMCI-trained nurses take care of some children, and as a result IMCI is only partially implemented. The study participants suggested that this challenge could be resolved by addressing the problem of understaffing, improving the patient–nurse ratio and advocating for more IMCI implementation resources.

### Challenges related to training coverage

Low IMCI training coverage of registered nurses posed a challenge, which was attributed to inadequate funding and the high cost of IMCI training courses. The study participants suggested that this challenge could be addressed by increasing the number of health workers trained in the IMCI strategy (i.e. scaling up both in-service and pre-service IMCI training). The second recommendation was that IMCI training should be extended to lower-level cadres, such as healthcare assistants, to increase the number of health workers able to deliver IMCI services. However, the lower-level cadres can only assist with non-clinical aspects of the IMCI protocol, such as counselling the caregiver.

### Challenges related to health systems

Study participants indicated that the layout of the health facility was not conducive or fully equipped to support the practice of IMCI procedures. They recommended that the health system provide a spacious facility layout and give time to apply IMCI skills and procedures (i.e. observation of first-dose treatment and rehydration corners). Another health-related system was lack of support from supervisors and colleagues. Study participants proposed that IMCI training be extended to senior managers to boost their confidence in supervising IMCI implementers. Yet another challenge related to health systems was the lack of IMCI follow-up training and visits. Respondents recommended that the health system ensure follow-up training and supervision of IMCI implementers by IMCI facilitators. They asserted that follow-up visits were important, as they could help newly trained practitioners to transfer their new skills to practice.

### Challenges related to client expectations

Issues related to client expectations have been attributed to the lack of uniformity between the IMCI-trained and the non-IMCI-trained health workers, in their case management practices. Non-IMCI-trained nurses take less time because they use single-diagnosis approaches, which do not view the client holistically and do not consider health problems other than the reason for the visit. As a result, caregivers of under-five patients prefer to be seen by them. This defeats the entire purpose of introducing the integrated approach. Two recommendations were proposed to standardise the practices of all registered nurses who tend under-five patients. The first recommendation was that all nurses tending under-five patients be IMCI-trained. The second recommendation was that IMCI training be the main criterion for the allocation of daily duties and the deployment of nurses to duty stations. This measure would help to ensure that only IMCI-trained nurses tend patients under the age of 5 years.

The participants made relevant recommendations that could be applied to promote and improve the use of the IMCI strategy. Recommendations for improving the use of the strategy included adopting short-duration IMCI training courses, scaling up both pre-service and in-service training, and extending IMCI training to lower-level cadres, as well as addressing the challenges related to health systems and the unique features of the strategy.

## Conclusions

If adopted, the proposed measures could lead to the promotion and improvement of the use of the IMCI strategy. This improvement could, in turn, lead to a decrease in the current morbidity and mortality rates in children under the age of five in Botswana. Such a decrease would contribute to the realisation of the goals articulated in the third pillar of Botswana's Vision 2016, as well as in MDG4.

The recommendations, although originally crafted for Botswana, can be adapted and used in various regions and countries to enhance the implementation of the IMCI strategy. If these challenges could be addressed, then maybe our children will have an opportunity to play.
